# Loss of IL-34 Expression Indicates Poor Prognosis in Patients With Lung Adenocarcinoma

**DOI:** 10.3389/fonc.2021.639724

**Published:** 2021-07-16

**Authors:** Zhendong Wang, Jun Zhu, Tianyi Wang, Hao Zhou, Jinjie Wang, Zhanghao Huang, Haijian Zhang, Jiahai Shi

**Affiliations:** ^1^ Nantong Key Laboratory of Translational Medicine in Cardiothoracic Diseases, and Research Institution of Translational Medicine in Cardiothoracic Diseases, Affiliated Hospital of Nantong University, Nantong, China; ^2^ Department of Thoracic Surgery, Affiliated Hospital of Nantong University, Nantong, China; ^3^ Research Center of Clinical Medicine, Affiliated Hospital of Nantong University, Nantong, China

**Keywords:** lung adenocarcinoma, interleukin 34, The Cancer Genome Atlas, prognosis, tissue microarray

## Abstract

Interleukin 34 (IL-34), an additional ligand of the colony-stimulating factor-1 receptor (CSF-1R), promotes the secretion of pro-inflammatory cytokines and stimulates NF-κB and JNK-related signaling pathways. However, the potential mechanism and prognostic value of IL-34 in lung adenocarcinoma (LUAD) remain obscure. In this study, IL-34 was found to be downregulated in LUAD tissues compared with para-carcinoma tissues, and loss of IL-34 expression was correlated with shorter overall survival (OS), which was validated by bioinformatics\ analysis in TCGA (The Cancer Genome Atlas) cohort and immunohistochemical analysis in the NTU (Nantong University) cohort, respectively. Subsequently, loss of IL-34 promotes negative regulation of the immune system and inhibits the infiltration of immune cells. Moreover, IL-34 deficiency was shown to be an independent adverse prognostic factor for patients with LUAD, and subgroup analysis indicated that IL-34 might contribute to the stratified management of patients with LUAD. IL-34-based nomogram model significantly improved the accuracy of prognostic predictions for OS of patients with LUAD, both in the TCGA cohort and the NTU cohort. Taken together, our data suggested that loss of IL-34 expression is associated with poor prognosis and negative regulation of the immune system of patients with LUAD, contributing to the stratified management of patients with LUAD.

## Introduction

Lung cancer is the most common cause of cancer-related deaths worldwide. Despite advances over the past decade in new targeted therapies, diagnostics, staging, and surgical techniques, as well as new chemotherapy and radiation therapy, lung cancer mortality remains high (1). Lung cancer mainly includes non-small cell lung cancer, which includes squamous cell carcinoma, adenocarcinoma, and large cell carcinoma; among them, adenocarcinoma accounts for most cases (2). The five-year overall survival (OS) rate of patients with lung adenocarcinoma (LUAD) is less than 15% because most patients are diagnosed as late-stage at the first visit (3). Therefore, it is crucial to determine suitable molecular markers for the early diagnosis and prognosis of LUAD (4).

Interleukin 34 (IL-34) is a cytokine that shares a common receptor with colony-stimulating factor (CSF)-1, namely CSF-1R (5). Previous studies have shown that IL-34 helps the development and maintenance of Langerhans cells and microglia (6, 7). IL-34 can play a role in modulating immunity in some autoimmune diseases, such as rheumatoid arthritis and systemic lupus erythematosus (8–10). IL-34 promotes the secretion of pro-inflammatory cytokines and stimulates NF-κB and JNK-related signaling pathways (11, 12). IL-34 can regulate the differentiation of macrophages and plays an important role in inflammatory diseases (13, 14). Macrophages are an important part of the tumor microenvironment; therefore, regulation by IL-34 may have a certain effect on tumor microenvironment changes (15). In 2016, research by Baghdadi et al. (16) showed that IL-34 enhanced the immunosuppressive effect of tumor-associated macrophages and mediated the survival of chemoresistant lung cancer cells. IL-34 has also been confirmed to be associated with the progression of ovarian cancer (17). IL-34 can promote cancer in a variety of solid tumors such as gastric cancer, colorectal cancer, liver cancer, and breast cancer (18–21). In addition to promoting tumor metastasis, IL-34 can also inhibit tumor differentiation and migration. IL-34 can inhibit the proliferation of glioblastoma cells, promote the differentiation of nonleukemia cells into monocyte-like cells, and inhibit the migration of HER2-positive breast cancer cells (22–25).

Although lung cancer has a high fatality rate, and IL-34 plays an important role in tumors, there are few studies on IL-34 in lung cancer. To the best of our knowledge, this is the first study to evaluate the prognostic value of IL-34 in LUAD, explore the relationship between IL-34 deficiency and immune cell infiltration and even immune negative regulation, finally established the nomogram model integrated IL-34 group and pathological characteristics to accurately predict the prognosis of patients with LUAD.

## Materials and Methods

### The Cancer Genome Atlas and Nantong University Cohorts

Gene expression data of LUAD patients in FPKM (Fragments Per Kilobase of transcript per Million mapped reads) form and corresponding clinical data were downloaded from TCGA database (https://tcga-data.download.nci.nih.gov/). Patients who lacked clinical information were excluded, and the gene expression values of the duplicate samples were replaced by their average values. Finally, 500 tumor samples of LUAD patients were incorporated in this study in addition to 59 normal samples and dichotomized into IL-34 high and IL-34 low groups according to the median of IL-34 mRNA.

The NTU cohort included 140 patients with LUAD who were confirmed by postoperative pathology and who had not received other treatments before surgery. Between 2009 and 2011, after surgery in our hospital, 140 LUAD and 34 non-tumor (normal) samples were collected from these patients. Moreover, these samples were all from different (unpaired) individuals. Patient clinical data were retrieved from the hospital’s records, including gender, smoking status, age, and tumor lymph node metastasis (TNM) stage. All patients were strictly staged according to TNM 8^th^ edition lung cancer staging.

### Tissue Microarray, Immunohistochemistry Staining, and Assessment of Immunohistochemistry Staining Intensity

A circular tissue with a diameter of 2 mm was removed from each tissue specimen, and the selected tissue cylinder was prepared as a tissue microarray specimen using the Unitma Quick-Ray tissue microarray analyzer (UT06; Unitma, Seoul, South Korea). Then, the tissue microarray specimen was sliced into 4 μm-thin tissue sections and arranged into microscope slides. Immunohistochemistry (IHC) staining was performed on the tissue chip using anti-IL-34 antibodies (ab224734; Abcam, Cambridge, MA, USA) as the primary antibody (1:200 dilution). Two professional pathologists assessed the percentage of IL-34-positive samples and the intensity of IL-34 IHC staining using a semi-quantitative immune response scoring system as the evaluation standard; the sample details were not revealed to them (26). The following formula was used to calculate the semi-quantitative H-score (0–300): IHC score = staining intensity value × positive percentage.

### Bioinformatics and Genetic Analyses

We further assessed the infiltration of immune cells in tumor tissues using cell-type identification by estimating relative subsets of RNA transcripts (CIBERSORT) (27), a deconvolution method based on a gene expression matrix. The number of random sample permutations was defined as 100. Single sample Gene Set Enrichment Analysis (ssGSEA) was used to quantify the enrichment levels of 29 immune-related gene sets in each sample according to the “GSVA” package. 29 immune signatures, which represented multiple immune cell types, functions, and pathways, were obtained from **Supplementary Table 1** (28). Then, we performed hierarchical clustering of LUAD samples, based on the enrichment levels (ssGSEA scores), as well as pathological characteristics including age, gender, TNM stage, T, N, M, and survival state. CIBERSORT analysis and ssGSEA analysis were carried out according to the gene expression matrix.

Subsequently, differential genes were selected according to the following criteria: P-value < 0.05. Gene symbols of these differential genes were converted into gene id for Gene Ontology (GO) and Kyoto Encyclopedia of Genes, Genomes (KEGG) and Gene Set Enrichment Analysis (GSEA) analysis according to the “clusterProfiler” package.

### Statistical Analysis

TCGA cohort was dichotomized into the IL-34 low group and the IL-34 high group according to the median of IL-34 mRNA expression. The NTU cohort was dichotomized into the IL-34 low group and the IL-34 high group according to 150 scores. Continuous variables were compared using the t-test or Wilcoxon rank-sum test. The Cox regression model was established to perform the univariate and multivariate analyses, and the characteristics with the statistical difference in the univariate analysis were further assessed by the multivariate analysis. Survival curves were created using the Kaplan–Meier method, and the difference between groups was determined by the log-rank analysis. The Nomogram model was established according to the “rms” package. P< 0.05 was considered statistically significant and all significance tests were two-sided (29).

### Ethics Approval and Consent to Participate

Each patient provided signed written informed consent. This retrospective study was authorized by the Clinical Research Ethics Committee of the Affiliated Hospital of Nantong University, Jiangsu, China.

## Results

### IL-34 Expression and Its Relationship With OS of Patients With LUAD

**Figure 1:**
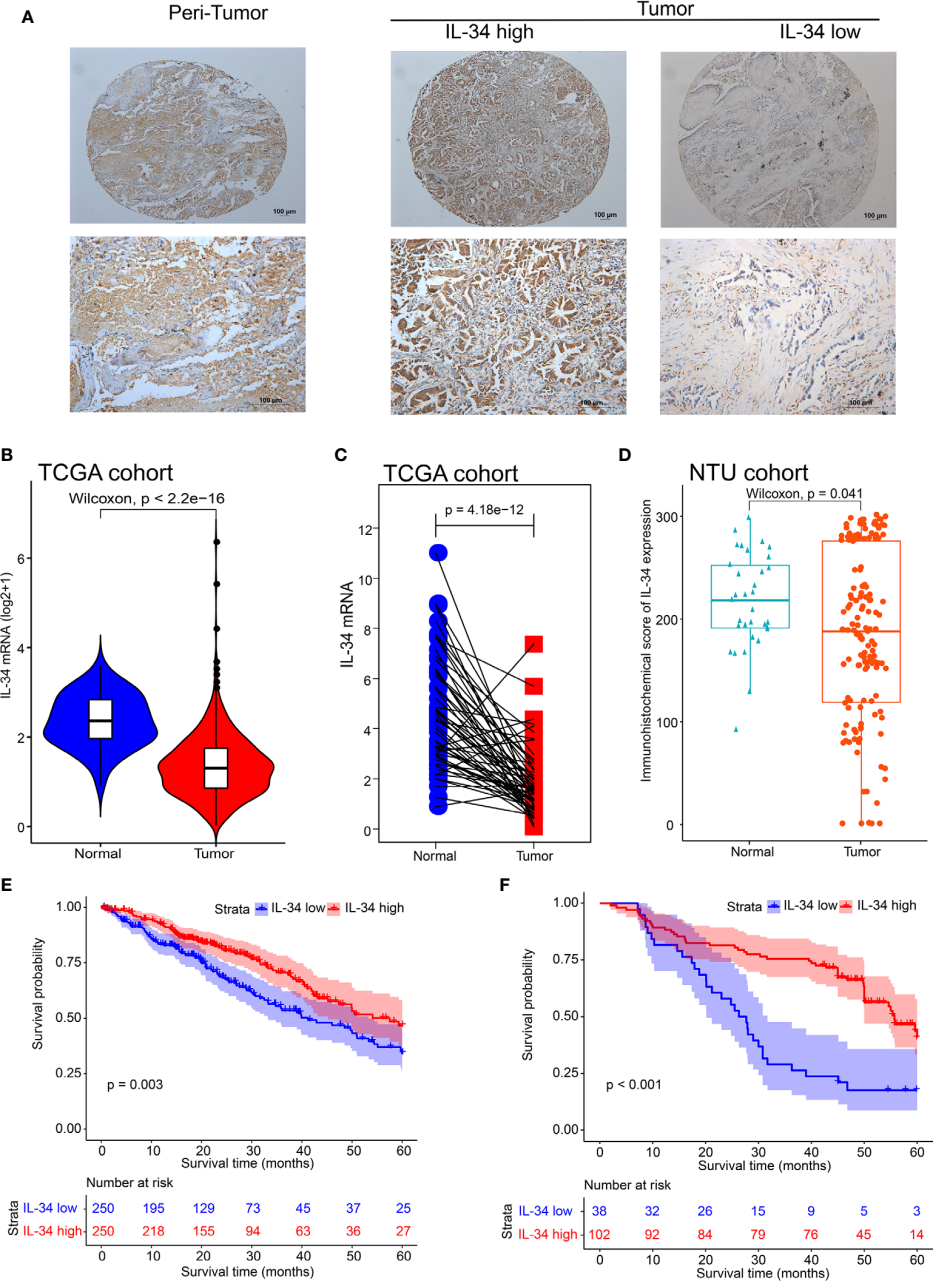
The expression deficiency of IL-34 and its correlation with prognosis overall survival (OS) in patients with lung adenocarcinoma (LUAD). **(A)** The typical image of immunohistochemistry for IL-34. **(B)** The expression of IL-34 in normal and tumor tissues in The Cancer Genome Atlas (TCGA) cohort. **(C)** Matching test of IL-34 expressed in TCGA cohort. **(D)** An immunohistochemical score of IL-34 expression in Nantong University (NTU) cohort. **(E)** Survival analysis between IL-34 high group and IL-34 low group in TCGA cohort. **(F)** Survival analysis between IL-34 high group and IL-34 low group in NTU cohort.

First, we performed IHC staining of the tumor tissues and adjacent non-tumor tissues obtained from 140 patients. Typical images of IL-34 IHC staining in LUAD and adjacent tissues are shown in **Figure 1A**. Next, we analyzed the mRNA level of IL-34 in samples from the TCGA database. Interestingly, the average expression level of IL-34 in tumor samples was lower than that in samples around the tumors (*P* < 0.001; **Figure 1B**). The expression levels of IL-34 in paired cancer and normal samples further confirm these results (*P* < 0.001; **Figure 1C**). Consistent with the data from TCGA, the expression of IL-34 in the cancer samples was lower than that in the normal samples (*P* < 0.001; **Figure 1D**). Moreover, in the TCGA cohort, the OS of patients with high IL-34 expression was longer than that of patients with low IL-34 expression (*P* = 0.003; **Figure 1E**). Similarly, in the NTU cohort (IHC set), patients with high IL-34 expression in tumors survived longer (*P* < 0.001; **Figure 1F**). The clinicopathological characteristics of patients with LUAD are listed in **Table 1** (TCGA cohort) and **Table 2** (the NTU cohort). Clinical information and the IHC score for IL-34 are shown in **Supplementary Table 1**.

**Table 1:** The clinicopathologic characteristics of patients with lung adenocarcinoma in TCGA cohort.

Characters	IL-34 low	IL-34 high	Total (n = 500)	P-value
	(n = 250)	(n = 250)		
Age				
≤65	118 (47.2%)	119 (47.6%)	237 (47.4%)	
>65	128 (51.2%)	125 (50%)	253 (50.6%)	
NA	4 (1.6%)	6 (2.4%)	10 (2%)	0.803
Gender				
Female	116 (46.4%)	154 (61.6%)	270 (54%)	
Male	134 (53.6%)	96 (38.4%)	230 (46%)	<0.001
Stage				
Stage I	123 (49.2%)	145 (58%)	268 (53.6%)	
Stage II	62 (24.8%)	57 (22.8%)	119 (23.8%)	
Stage III	47 (18.8%)	33 (13.2%)	80 (16%)	
Stage IV	15 (6%)	10 (4%)	25 (5%)	
NA	3 (1.2%)	5 (2%)	8 (1.6%)	0.178
T				
T1	60 (24%)	98 (39.2%)	158 (31.6%)	
T2	143 (57.2%)	103 (41.2%)	246 (49.2%)	
T3	24 (9.6%)	15 (6%)	39 (7.8%)	
T4	11 (4.4%)	7 (2.8%)	18 (3.6%)	
NA	12 (4.8%)	27 (10.8%)	39 (7.8%)	<0.001
M				
M0	165 (66%)	147 (58.8%)	312 (62.4%)	
M1	14 (5.6%)	9 (3.6%)	23 (4.6%)	
NA	71 (28.4%)	94 (37.6%)	165 (33%)	0.07
N				
N0	150 (60%)	151 (60.4%)	301 (60.2%)	
N1	46 (18.4%)	40 (16%)	86 (17.2%)	
N2	39 (15.6%)	25 (10%)	64 (12.8%)	
N3	0 (0%)	2 (0.8%)	2 (0.4%)	
NA	15 (6%)	32 (12.8%)	47 (9.4%)	0.014

ACT, adjuvant chemoradiotherapy; TNM, tumor-node-metastasis; NA, not applicable.

**Table 2:** The clinicopathologic characteristics of patients with lung adenocarcinoma in the Nantong University (NTU) cohort.

Characters	IL-34 low	IL-34 high	Total (n = 140)	P-value
	(n =38)	(n = 102)		
**sex**				
sex_0	13 (34.2%)	39 (38.2%)	52 (37.1%)	
sex_1	25 (65.8%)	63 (61.8%)	88 (62.9%)	0.809
**age**				
<=65	13 (34.2%)	48 (47.1%)	61 (43.6%)	
>65	25 (65.8%)	54 (52.9%)	79 (56.4%)	0.241
**smoke**				
No	29 (76.3%)	77 (75.5%)	106 (75.7%)	
Yes	9 (23.7%)	25 (24.5%)	34 (24.3%)	1
**TNM**				
Stage I	20 (52.6%)	55 (53.9%)	75 (53.6%)	
Stage II	10 (26.3%)	29 (28.4%)	39 (27.9%)	
Stage III	7 (18.4%)	17 (16.7%)	24 (17.1%)	
Stage IV	1 (2.6%)	1 (1%)	2 (1.4%)	0.987
**ACT**				
Without	20 (52.6%)	40 (39.2%)	60 (42.9%)	
With	18 (47.4%)	62 (60.8%)	80 (57.1%)	0.217

ACT, adjuvant chemoradiotherapy; TNM, tumor-node-metastasis.

### Univariate and Multivariate Analyses

**Figure 2:**
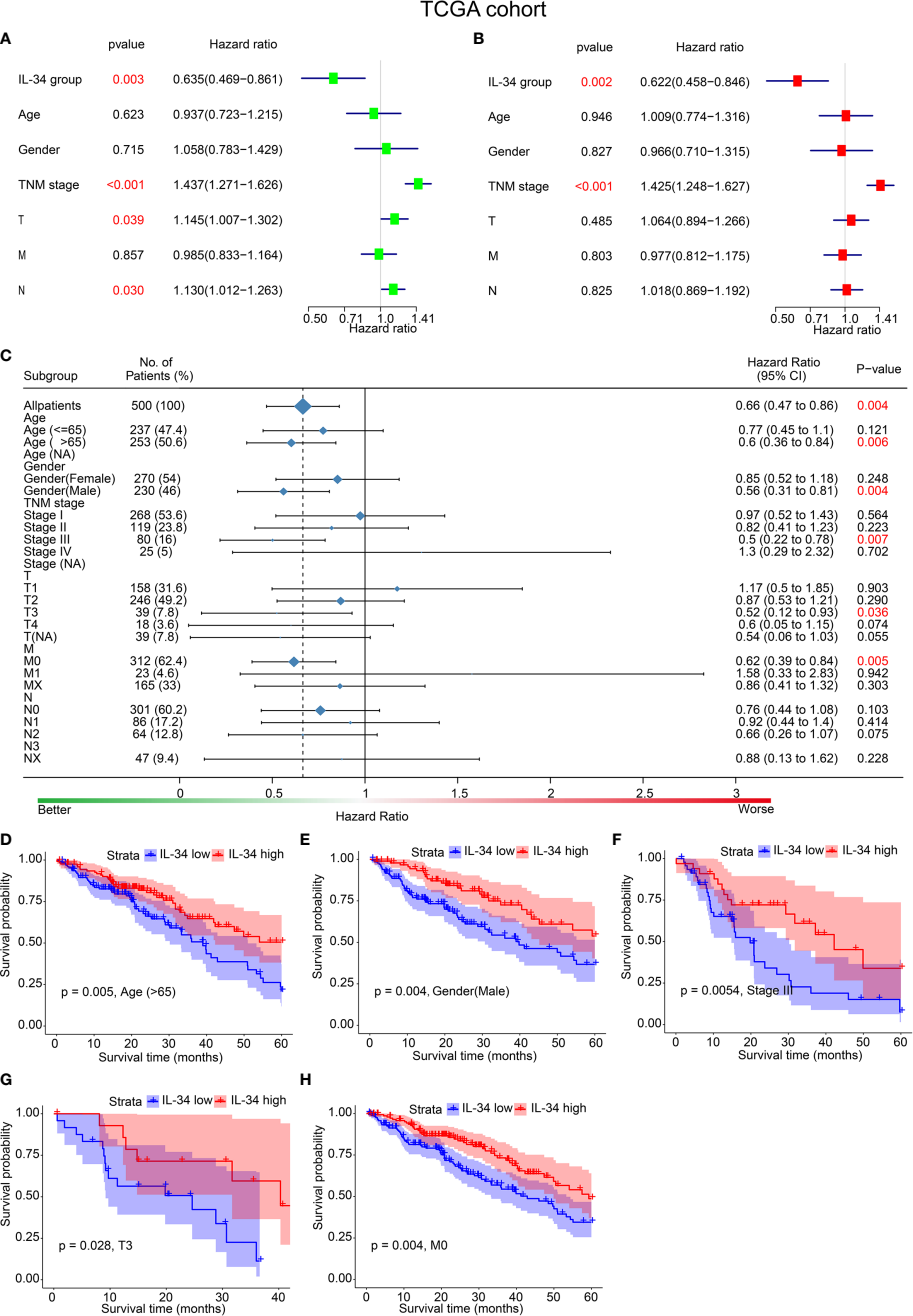
IL-34 was an independent protective factor of lung adenocarcinoma (LUAD) in The Cancer Genome Atlas (TCGA) cohort. **(A)** Univariate analysis for overall survival in TCGA cohort. **(B)** Multivariate analysis for overall survival in TCGA cohort. **(C)** Subgroup analysis between IL-34 high group and IL-34 low group based on Cox regression. **(D–H)** Survival analysis for significant clinicopathologic features between IL-34 high group and IL-34 low group.

The results of both univariate and multivariate analyses in the TCGA cohort are summarized in **Figure 2**. In the univariate analysis, IL-34 expression (*P* = 0.003) and TNM stage (*P* < 0.001) were significant risk factors for OS (**Figure 2A**). Similarly, IL-34 expression (*P* = 0.002) and TNM stage (*P* < 0.001) were significant risk factors for OS in the multivariate analysis (**Figure 2B**). Subsequently, the patients were divided into different subgroups, according to clinicopathological characteristics. Based on the results of the analysis, the relationship between IL-34 expression and patient OS was exhibited as a forest plot. The results of subgroup analysis further showed that loss of IL-34 expression was related to poor prognosis for LUAD patients who were older than 65 (*P* = 0.006), male (*P* = 0.004), in stage III (*P* = 0.007), with tumor diameter T3 (*P* = 0.036), or M0 (No distant metastasis, *P* = 0.005). According to the difference in IL-34 expression, the survival analysis showed that high IL-34 expression was associated with a longer OS of LUAD patients who were older than 65, male, or with TNM stage III, T3, or M0 (**Figures 2D–H**).

**Figure 3:**
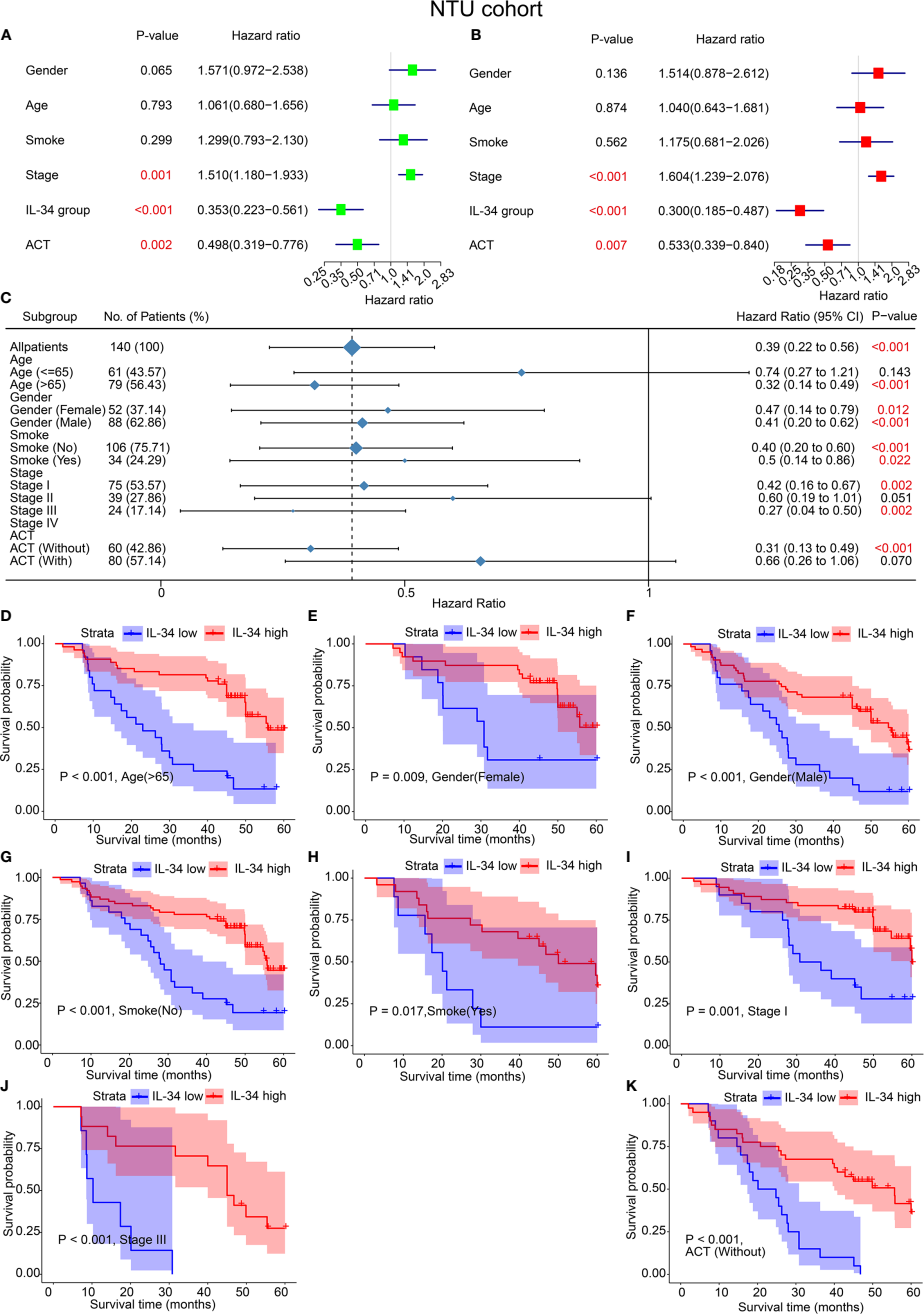
IL-34 was an independent protective factor of lung adenocarcinoma (LUAD) in the Nantong University (NTU) cohort. **(A)** Univariate analysis for overall survival in the NTU cohort. **(B)** Multivariate analysis for overall survival in the NTU cohort. **(C)** Subgroup analysis between IL-34 high group and IL-34 low group based on Cox regression. **(D–K)** Survival analysis for significant clinicopathologic features between IL-34 high group and IL-34 low group.

Analogously, the results of both univariate and multivariate analyses of the IHC set are shown in **Figure 3**. In the univariate analyses, IL-34 expression (*P* < 0.001), adjuvant chemoradiotherapy (ACT, *P* = 0.002), and TNM stage (*P* = 0.001) were significant risk factors for OS (**Figure 3A**). Multivariate analysis results were the same as the univariate analysis results (**Figure 3B**). The results of subgroup analysis further showed that loss of IL-34 expression was related to poor prognosis for LUAD patients who were older than 65 (*P* < 0.001), female (*P* = 0.012), male (*P* < 0.001), non-smokers (*P* < 0.001), smokers (*P* = 0.022), in stage I (*P* = 0.002) or stage III (*P* = 0.002), or had not received ACT (*P* < 0.001, **Figure 3C**). According to the difference in IL-34 expression, the survival analysis showed that high IL-34 expression was associated with a longer OS of LUAD patients who were older than 65, male, female, smokers, non-smokers, in TNM stage I or TNM stage III, or had not received ACT (**Figures 3D–K**).

Strikingly, the majority of patients with LUAD were early-stage patients with TNM stage I or II, in both the TCGA cohort (77.40%) and the NTU cohort (81.43%). However, a significant survival difference of early-stage patients between the IL-34 high group and IL-34 low group was only found in the NTU cohort but not in the TCGA cohort, which was a striking inconsistency (**Supplementary Figure 1**).

### Correlation Analysis of IL-34 and Immune Cell Infiltration

**Figure 4:**
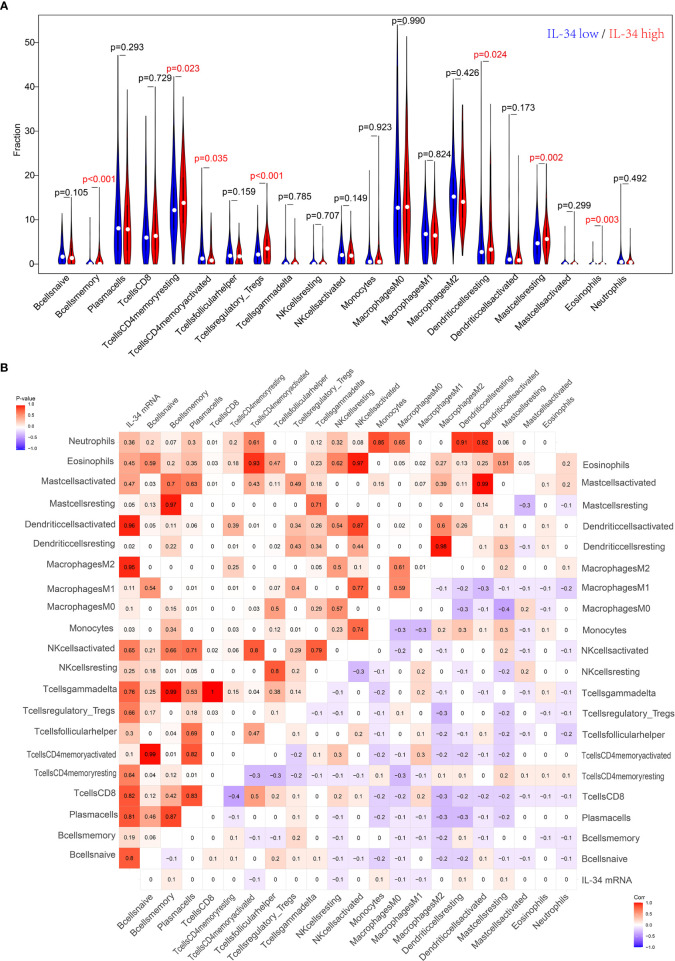
Loss of IL-34 inhibited the immune cell infiltration. **(A)** Immune cells infiltration between IL-34 high group and IL-34 low group. **(B)** Correlation analysis between the expression of IL-34 mRNA and immune cells.

IL-34 promotes the secretion of pro-inflammatory cytokines and stimulates NF-κB and JNK-related signaling pathways (11, 12); hence, we wanted to explore the relationship between IL-34 and tumor immune cell infiltration. Based on the difference in IL-34 expression, the gene expression profile of TCGA cases was classified and shown by heatmap and volcano map in **Supplementary Figure 2**. As shown in **Figure 4A**, we found that the fraction of immune cells was different between IL-34 high group and IL-34 low group in resting Mast cells, resting CD4^+^ memory T cells, resting dendritic cells, activated CD4^+^ memory T cells, regulatory T cells, memory B cells, and eosinophils. In the tumor tissues of patients with high IL-34 expression, the proportion of these immune cells was higher except for that of activated CD4^+^ memory T cells. However, naive B cells, plasma cells, CD8^+^ T cells, follicular helper T cells, and other immune cells were not changed between the two groups (**Figure 4A**). We also evaluated the possible correlations between 21 immune cells and IL-34 expression (**Figure 4B**). The generated heatmap shows that the ratio of the tumor-infiltrating immune cell subsets had some degree of correlation. For example, IL-34 was positively related to monocytes and resting dendritic cells. Therefore, the variations in the infiltration proportion suggested that IL-34 promotes infiltration of immune cells. Conversely, IL-34 deficiency inhibits the infiltration of immune cells.

### Relationship Between Clinicopathological Characteristics and Tumor Immune Infiltration

**Figure 5:**
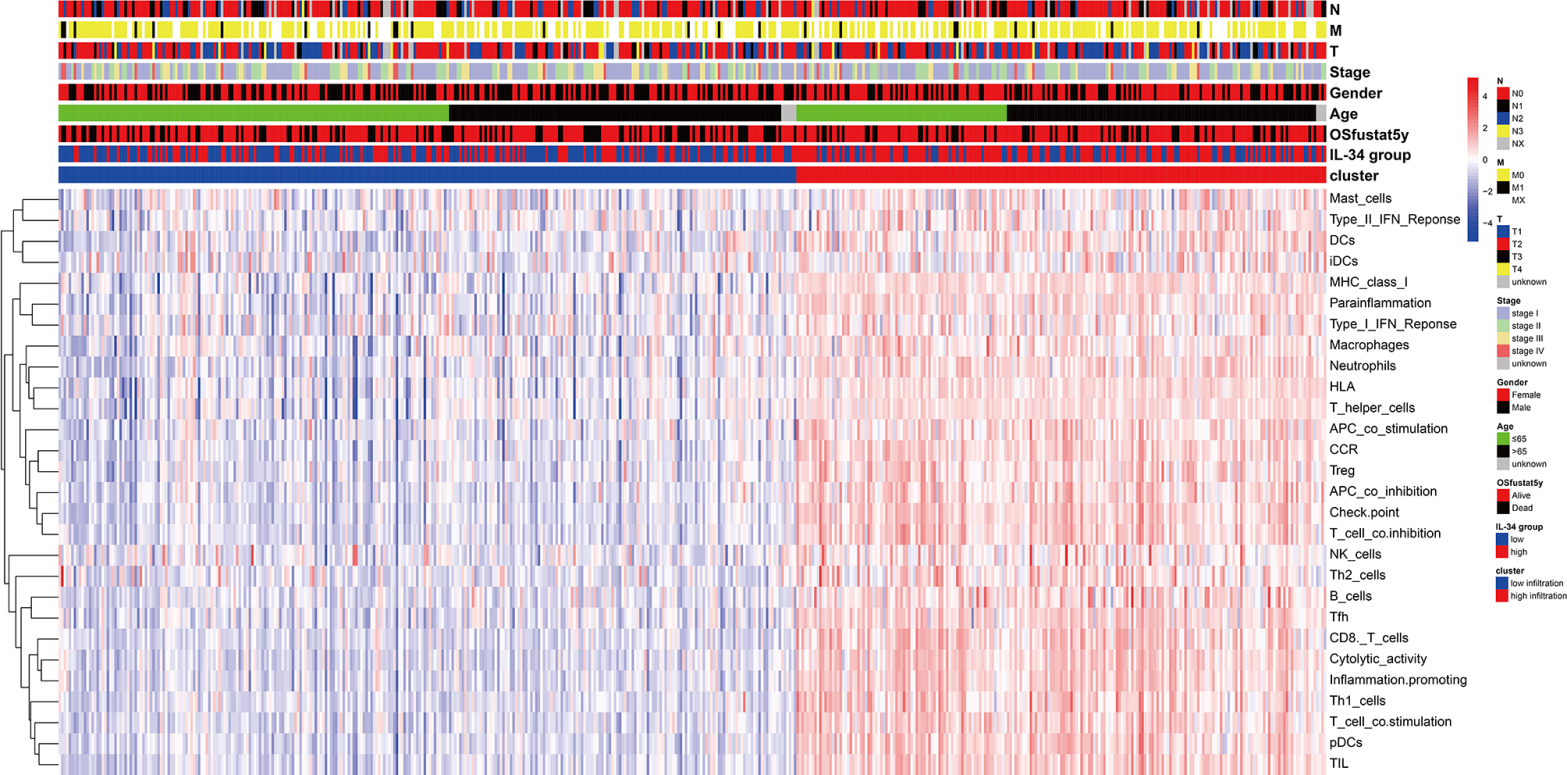
Heatmap of pathological characteristics and 29 immune-related gene sets.

We prepared a heatmap based on the integration of pathological characteristics and 29 immune-related gene sets, which represented multiple immune cell types, functions, and pathways (**Figure 5**). Most samples with high IL-34 expression showed higher signature scores of immune-related gene sets and a longer OS period. This may be the result of immune cells functioning in tumor tissue. Therefore, IL-34 deficiency inhibits the infiltration of immune cells.

### IL-34 Was Associated With Immune Response and Inflammation-Related Signaling Pathways

**Figure 6:**
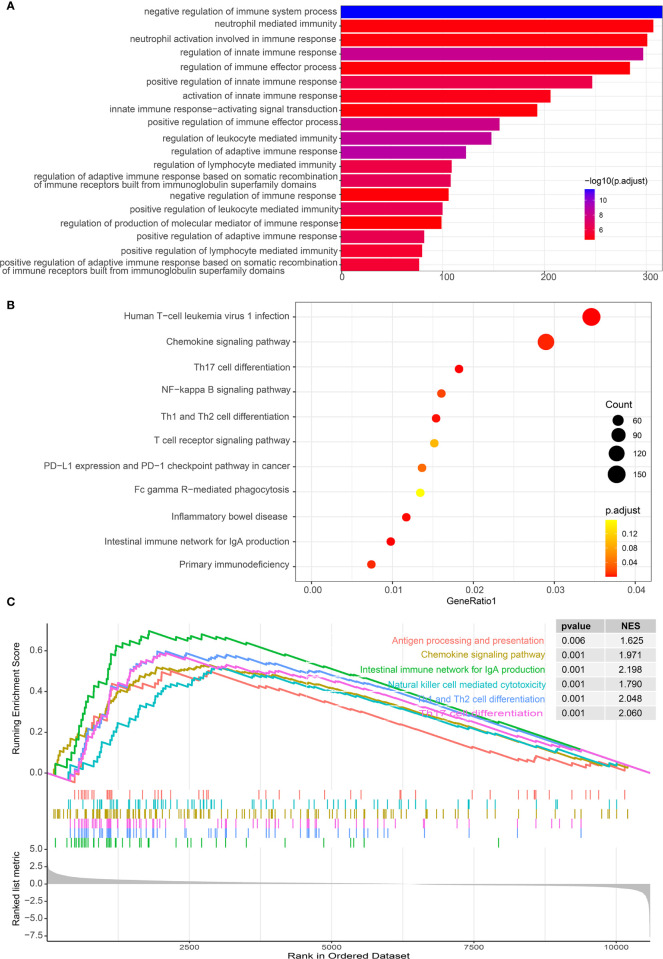
Functional analysis of differential genes between IL-34 high group and IL-34 low group. **(A)** Gene Ontology (GO) analysis. **(B)** Kyoto Encyclopedia of Genes and Genomes (KEGG) analysis. **(C)** Gene set enrichment analysis (GSEA) analysis.

As IL-34 is related to tumor immune cells, we performed GO/KEGG/GSEA to explore whether pathways related to tumor immunity were enriched. As expected, GO enrichment analysis showed that IL-34 was mainly involved in immune regulatory activities, such as “negative regulation of immune system process”, “neutrophil-mediated immunity”, and “neutrophil activation involved in immune response” (**Figure 6A**). Pathways generated by KEGG analysis are shown in **Figure 6B**. The top 10 pathways were “human T-cell leukemia virus 1 infection”, “chemokine signaling pathway”, “Th17 cell differentiation”, “NF-kappa B signaling pathway”, “Th1 and Th2 cell differentiation”, “T cell receptor signaling pathway”, “PD-L1 expression” and “PD-1 checkpoint pathway in cancer”, “Fc gamma R-mediated phagocytosis”, “inflammatory bowel disease”, and “intestinal immune network for IgA production”. In addition, a GSEA map was also generated based on differential genes. Notably, GSEA revealed that the gene sets with high scores were immune-related pathways, such as “Antigen processing and presentation”, “Chemokine signaling pathway”, “Natural killer cell-mediated cytotoxicity” and “Th17 cell differentiation” (**Figure 6C**).

### Prognostic Nomograms for OS of Patients with LUAD

**Figure 7:**
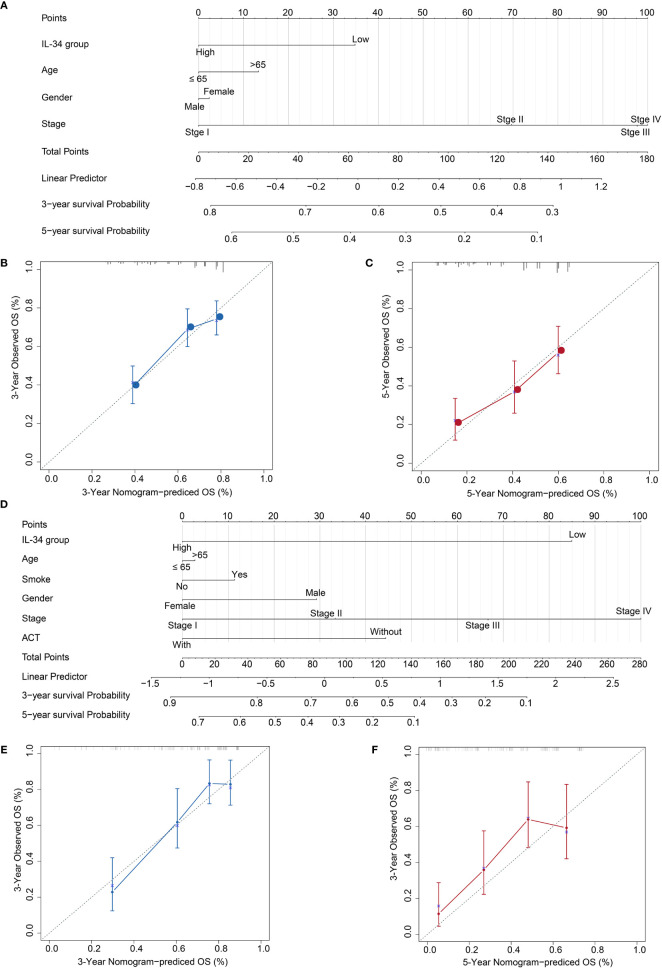
Calibration plot of the three- and five-year overall survival (OS) nomogram in The Cancer Genome Atlas (TCGA) cohort and the Nantong University (NTU) cohort. **(A)** The nomogram consists of the signature score based on the expression of IL-34, age, gender, and stage in the TCGA cohort. **(B)** Calibration curve for 3-year OS in TCGA cohort. **(C)** Calibration curve for 5-year OS in TCGA cohort. **(D)** The nomogram consists of the signature score based on the expression of IL-34, smoke, age, gender, and stage in the NTU cohort. **(E)** Calibration curve for 3-year OS in the NTU cohort. **(F)** Calibration curve for 5-year OS in the NTU cohort.

Based on the results, we established a three-year and five-year survival prognosis model for patients with LUAD (**Figure 7**). For the values attributed to each risk factor, the patients received points, and the total points (i.e., the sum of the points received for each risk factor) were used to predict the OS. Therefore, a higher total score was associated with a lower OS (**Figures 7A, D**). The calibration graph shows that the curves of actual OS and predicted OS is highly fitted, indicating that our nomogram predictions performed well, both in the TCGA cohort (3-year OS, **Figure 7B**; 5-year OS, **Figure 7C**) and the NTU cohort (3-year OS, **Figure 7E**, 5-year OS, **Figure 7F**). To compare the advantages of the nomogram model over traditional prognostic indicators in predicting OS, we analyzed the accuracy of the nomogram model and TNM stage in predicting 5-year OS. As shown in IL-34 **Supplementary Figure 3**, the area under the curve (AUC) of the nomogram model was improved compared with that of the TNM stage, as well as the IL-34 group, both in the TCGA cohort (**Supplementary Figure 3A**) and NTU cohort (**Supplementary Figure 3B**).

## Discussion

Studies have shown that lung cancer is the leading cause of cancer-related deaths worldwide among all types of cancer (30). There were 1.76 million lung cancer deaths in 2018, accounting for nearly one-fifth of all cancer deaths (31, 32). Several studies have confirmed that IL-34 is related to tumors, but few studies have investigated IL-34 in LUAD (15, 18, 20). Thus, we conducted a study based on existing data to explore the prognostic value of IL-34 in patients with LUAD. In this study, IL-34 expression was found to be significantly correlated with OS of LUAD patients, especially in patients in clinical stage III. Patients with high IL-34 expression showed longer OS than those with low IL-34 expression.

IL-34 was first discovered in 2008, and it shares a common receptor with CSF-1, namely CSF-1R (5, 33). IL-34 can recruit tumor-associated macrophages, which drive the formation of new blood vessels in cancer; hence, IL-34 is assumed to promote tumor progression (34). IL-34 is thought to mediate the induced survival of lung cancer cells, and hence may be disadvantageous to patients (16). In 2018, Baghdadi et al. found that chemotherapy-induced IL-34 enhances immunosuppression by tumor-associated macrophages and mediates survival of chemoresistant lung cancer cells, and the patients with lung cancer have a shorter OS in the IL-34 high group compared with that in IL-34 low group, which is opposite to our finding (16). The reasons behind the phenomenon may lie in two aspects: Firstly, the lung cancer samples they used were not just lung adenocarcinoma, but also squamous cell and small-cell lung cancers. In fact, our samples, both the TCGA data and our immunohistochemical samples, are all lung adenocarcinoma samples. Secondly, there was a significant data skew in their IL-34 high-low grouping, with 249 IL-34 low-expressed samples and only 83 IL-34 high-expressed samples. We used a more scientific median value as the threshold in the TCGA cohort, so the results are more reliable. As for grouping in the NTU cohort, we chose the mean of full scores as the threshold, given the small sample size.

The existing literature reports show that IL-34 may promote the differentiation of monocytes into macrophages in some diseases, and the expression of IL-34 is related to M2 macrophages (15, 35). In our study, immune microenvironment analysis and GO and KEGG enrichment analyses showed that IL-34 expression was closely related to tumor immune responses and inflammation-related signaling pathways, which is consistent with the results from previous studies (5, 13, 17, 35, 36). IL-34 expression is significantly related to natural killer (NK) cells, M2 macrophages, and memory B cells. IL-34 and M-CSF can induce macrophages to differentiate into the M2 type, leading to immune suppression mediated by M2 macrophages (37). It is worth noting that M2 macrophages are generally considered cancer-promoting, which seems to differ from our results, and the reasons for this need to be explored (38, 39). IL-34 is related to PD-L1 expression and the PD-1 signaling pathway. In early lung cancer, high PD-L1 expression status represents a biomarker that can signify a good prognosis after radical surgery (40).

Collectively, these results suggest that IL-34 expression could have important clinical significance for the prognosis of patients with LUAD. Further work is needed to strengthen the findings of our study. First, owing to the lack of more detailed information, this study only reports an analysis of the correlation between IL-34 expression and OS. Determining further relevance will require additional work, and our future research will focus on filling these gaps. Second, the prognostic value of IL-34 expression in patients with LUAD should be further verified in more extensive multi-center clinical trials, which could improve the accuracy of the prediction model. Finally, the role of IL-34 in the occurrence and development of LUAD is not fully understood, especially since it exhibits different effects in early lung cancer and advanced lung cancer, which requires further research to explore the underlying mechanism.

In conclusion, this study determined that loss of IL-34 could predict poor prognosis in patients with LUAD and that examination of its expression level may improve postoperative grading management of patients based on the TNM stage. In addition, our established nomogram incorporating IL-34 expression and other risk factors shows a significant improvement in the accuracy of patient survival prediction, thus contributing to precision medicine.

## Data Availability Statement

The datasets presented in this study can be found in online repositories. The names of the repository/repositories and accession number(s) can be found in the article/**Supplementary Material**.

## Ethics Statement

The studies involving human participants were reviewed and approved by Clinical Research Ethics Committee of the Affiliated Hospital of Nantong University. The patients/participants provided their written informed consent to participate in this study. Written informed consent was obtained from the individual(s) for the publication of any potentially identifiable images or data included in this article.

## Author Contributions

Study concept and design: HZha and JS. Technical and material support: ZW, JZ, TW, HZho, JW, and ZH. Analysis and interpretation of data and drafting of the manuscript: ZW and JZ. Obtained funding and study supervision: HZha and JS. All authors contributed to the article and approved the submitted version.

## Funding

This work was supported by funds from the National Natural Science Foundation of China (81770266), the “Six-one” Project for High-level Health Talents of Jiangsu province, China (LGY2016037), Nantong Key Laboratory of Translational Medicine in Cardiothoracic Diseases, Nantong Clinical Medical Research Centre of Cardiothoracic Disease, and Institution of Translational Medicine in Cardiothoracic Diseases in Affiliated Hospital of Nantong University.

## Conflict of Interest

The authors declare that the research was conducted in the absence of any commercial or financial relationships that could be construed as a potential conflict of interest.

## Abbreviations

IL-34, interleukin 34; LUAD, lung adenocarcinoma; TCGA, The Cancer Genome Atlas; OS, overall survival; TNM, tumor-node-metastasis; mRNA, messenger RNA; CSF, Colony-stimulating factor; IHC, immunohistochemistry; GO, gene ontology; KEGG, Kyoto Encyclopedia of Genes and Genomes; GSEA, Gene Set Enrichment Analysis; ACT, Adjuvant chemoradiotherapy; NTU, Nantong University.
