# Supplementary material for: Loss of IL-34 Expression Indicates Poor Prognosis in Patients With Lung Adenocarcinoma
Source: Front Oncol. 2021 Jul 16;11:639724. doi: 10.3389/fonc.2021.639724 (PMC8322957; doi:10.3389/fonc.2021.639724)
Supplement: Supplementary file 1 [file DataSheet_1.docx]

Supplementary Material

## Supplementary Figures

**
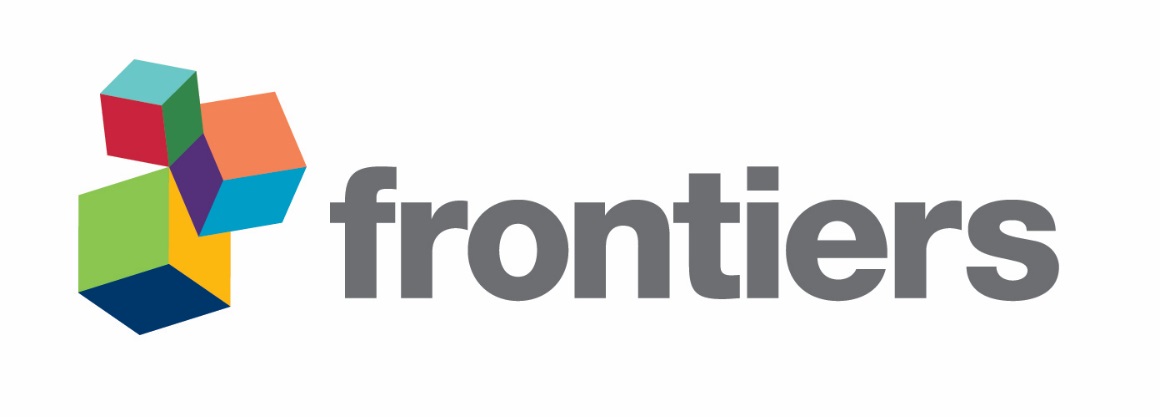
**

**Supplementary Figure 1.** Survival curves of patients with early lung adenocarcinoma (TNM stage I or II) in TCGA (The Cancer Genome Atlas) cohort (**A**) and NTU (Nantong University) cohort (**B**).

**Supplementary Figure 2.** Heatmap (**A**) and volcano plot (**B**) of differentially expressed genes between the different IL-34 expression groups.

**Supplementary Figure 3.** The 5-year receiver operating curve (ROC) curve in TCGA (The Cancer Genome Atlas) cohort (**A**) and NTU (Nantong University) cohort (**B**).

**Supplementary Tables**

**Supplementary Table 1.** Clinical information and immunohistochemical score of IL-34.

^#^, the age of the patients was recorded in years, and the corresponding dummy variables were constructed as follows: 0, ≤65 years old; 1, ＞ 65 years old. The dummy variables for gender as follows: 0, female; 1, male. The dummy variables for smoking as follows: 0, no smoking; 1, smoking. The dummy variables for death as follows: 0, alive; 1, death. The dummy variables for ACT (adjuvant chemoradiotherapy) as follows: 0, Without ACT; 1, with ACT.

| **Supplementary Table 1. Clinical information and immunohistochemical score of IL-34^#^.** | | | | | | | | | | | |
| --- | --- | --- | --- | --- | --- | --- | --- | --- | --- | --- | --- |
| Id | Survival | Death | Pathyology Id | Sex | Age | Smoke | TNM | IL34_low0_high1 | IL34 score | ACT | Location |
| p1 | 12.33 | 1 | 501269 | 0 | 0 | 0 | 1 | 1 | 247 | 1 | array_1B1 |
| p2 | 60 | 1 | 508800 | 1 | 0 | 1 | 1 | 1 | 287 | 0 | array_1G1 |
| p3 | 5 | 1 | 510128 | 1 | 1 | 0 | 1 | 1 | 152 | 1 | array_1C1 |
| p4 | 59.6 | 1 | 511496 | 1 | 0 | 1 | 1 | 1 | 222 | 1 | array_1E1 |
| p5 | 60 | 0 | 511596 | 1 | 1 | 1 | 2 | 1 | 295 | 0 | array_1F1 |
| p6 | 60 | 0 | 602683 | 1 | 1 | 1 | 2 | 1 | 282 | 1 | array_1B2 |
| p7 | 16.17 | 1 | 603258 | 1 | 0 | 0 | 3 | 1 | 281 | 0 | array_1C2 |
| p8 | 60 | 0 | 604009 | 1 | 0 | 1 | 3 | 1 | 275 | 0 | array_1D2 |
| p9 | 21.2 | 1 | 606099 | 1 | 1 | 1 | 4 | 0 | 96 | 1 | array_1F2 |
| p10 | 13.5 | 1 | 606674 | 1 | 0 | 1 | 1 | 1 | 163 | 1 | array_1D8 |
| p11 | 49.9 | 1 | 608851 | 0 | 0 | 0 | 3 | 1 | 151 | 0 | array_1A4 |
| p12 | 60 | 0 | 610137 | 1 | 0 | 0 | 3 | 1 | 205 | 0 | array_1C3 |
| p13 | 26.13 | 1 | 610256 | 1 | 0 | 0 | 2 | 1 | 180 | 0 | array_1C4 |
| p14 | 60 | 0 | 610257 | 1 | 1 | 1 | 2 | 1 | 280 | 1 | array_1D4 |
| p15 | 10 | 1 | 612151 | 1 | 0 | 0 | 2 | 1 | 296 | 1 | array_1D3 |
| p16 | 30 | 1 | 613062 | 1 | 1 | 1 | 2 | 0 | 122 | 1 | array_2B9 |
| p17 | 16.03 | 1 | 615540 | 1 | 1 | 1 | 2 | 1 | 292 | 0 | array_1E5 |
| p18 | 2.02 | 1 | 700041 | 1 | 1 | 0 | 2 | 1 | 249 | 0 | array_1D5 |
| p19 | 39.57 | 1 | 700861 | 0 | 1 | 1 | 2 | 1 | 156 | 0 | array_1B5 |
| p20 | 14.63 | 1 | 702391 | 0 | 1 | 0 | 2 | 0 | 79 | 0 | array_1F5 |
| p21 | 7.17 | 1 | 703206 | 1 | 1 | 0 | 3 | 0 | 119 | 0 | array_1G5 |
| p22 | 9.53 | 1 | 710917 | 0 | 0 | 0 | 2 | 1 | 278 | 1 | array_1B6 |
| p23 | 7.6 | 1 | 710919 | 1 | 1 | 1 | 2 | 0 | 79 | 0 | array_1D6 |
| p24 | 49.8 | 1 | 711015 | 0 | 1 | 0 | 1 | 1 | 174 | 1 | array_1E6 |
| p25 | 60 | 0 | 711602 | 1 | 1 | 0 | 1 | 1 | 173 | 0 | array_1F6 |
| p26 | 9.03 | 1 | 713262 | 1 | 1 | 0 | 1 | 0 | 0 | 0 | array_2C9 |
| p27 | 55.37 | 1 | 714856 | 1 | 1 | 0 | 3 | 1 | 188 | 1 | array_1A7 |
| p28 | 46.77 | 1 | 715083 | 1 | 0 | 1 | 3 | 1 | 217 | 1 | array_1B7 |
| p29 | 9.77 | 1 | 715564 | 1 | 1 | 0 | 1 | 0 | 53 | 0 | array_1E7 |
| p30 | 60 | 0 | 716597 | 0 | 1 | 0 | 1 | 1 | 278 | 1 | array_1A8 |
| p31 | 7.13 | 1 | 716598 | 0 | 0 | 0 | 3 | 1 | 276 | 1 | array_1F7 |
| p32 | 60 | 0 | 716850 | 1 | 0 | 1 | 1 | 1 | 179 | 0 | array_1G7 |
| p33 | 60 | 0 | 803709 | 1 | 1 | 0 | 1 | 1 | 290 | 1 | array_1B9 |
| p34 | 20.13 | 1 | 804451 | 0 | 0 | 0 | 2 | 0 | 119 | 0 | array_1E9 |
| p35 | 60 | 0 | 805861 | 0 | 0 | 0 | 1 | 1 | 219 | 0 | array_1G9 |
| p36 | 7.7 | 1 | 807054 | 1 | 1 | 0 | 2 | 1 | 223 | 0 | array_1A10 |
| p37 | 59.73 | 0 | 808410 | 1 | 1 | 0 | 1 | 1 | 232 | 0 | array_1B10 |
| p38 | 59.23 | 0 | 809285 | 1 | 1 | 0 | 1 | 1 | 277 | 0 | array_1D10 |
| p39 | 54.63 | 1 | 809594 | 1 | 0 | 0 | 1 | 1 | 168 | 1 | array_2A1 |
| p40 | 8.67 | 1 | 810762 | 1 | 1 | 1 | 3 | 0 | 124 | 1 | array_2B1 |
| p41 | 58.23 | 0 | 810901 | 1 | 1 | 1 | 2 | 1 | 276 | 1 | array_2C1 |
| p42 | 16.33 | 1 | 811461 | 1 | 1 | 1 | 1 | 1 | 164 | 1 | array_2F1 |
| p43 | 50 | 1 | 811776 | 1 | 1 | 1 | 1 | 1 | 299 | 1 | array_2G1 |
| p44 | 10.27 | 1 | 811973 | 0 | 1 | 0 | 3 | 0 | 119 | 1 | array_2D8 |
| p45 | 50 | 1 | 812752 | 1 | 1 | 0 | 2 | 1 | 287 | 1 | array_2C8 |
| p46 | 55.4 | 0 | 815101 | 1 | 0 | 0 | 2 | 1 | 156 | 1 | array_2G7 |
| p47 | 8.53 | 1 | 815177 | 1 | 1 | 0 | 3 | 0 | 117 | 1 | array_2A9 |
| p48 | 50 | 1 | 815646 | 1 | 0 | 0 | 2 | 1 | 277 | 1 | array_2F8 |
| p49 | 55 | 1 | 816113 | 0 | 0 | 0 | 2 | 1 | 203 | 1 | array_2A8 |
| p50 | 50 | 1 | 816409 | 0 | 1 | 0 | 1 | 1 | 187 | 1 | array_2E7 |
| p51 | 45 | 1 | 817729 | 1 | 1 | 0 | 3 | 1 | 200 | 0 | array_2B7 |
| p52 | 53.77 | 0 | 818472 | 0 | 0 | 0 | 4 | 1 | 150 | 0 | array_2B10 |
| p53 | 3.17 | 1 | 818978 | 1 | 0 | 1 | 1 | 1 | 296 | 0 | array_2G9 |
| p54 | 25.5 | 1 | 900643 | 1 | 0 | 0 | 2 | 1 | 180 | 0 | array_4A1 |
| p55 | 25.5 | 1 | 900643 | 1 | 0 | 0 | 2 | 0 | 80 | 0 | array_4B1 |
| p56 | 51.5 | 0 | 902248 | 1 | 1 | 1 | 1 | 1 | 279 | 1 | array_2F4 |
| p57 | 14.6 | 1 | 902250 | 1 | 1 | 0 | 2 | 1 | 157 | 0 | array_2E4 |
| p58 | 51.4 | 0 | 902466 | 1 | 1 | 0 | 2 | 1 | 280 | 1 | array_2C5 |
| p59 | 60 | 0 | 902469 | 0 | 0 | 0 | 2 | 0 | 78 | 1 | array_4A6 |
| p60 | 50 | 1 | 902890 | 0 | 0 | 0 | 1 | 1 | 291 | 1 | array_2D5 |
| p61 | 45.1 | 1 | 902982 | 1 | 1 | 0 | 1 | 0 | 20 | 0 | array_4E1 |
| p62 | 50.9 | 0 | 903136 | 0 | 1 | 0 | 1 | 1 | 217 | 1 | array_2E5 |
| p63 | 30.8 | 1 | 903299 | 0 | 0 | 0 | 3 | 0 | 97 | 0 | array_4F1 |
| p64 | 45 | 1 | 903517 | 1 | 1 | 0 | 3 | 1 | 278 | 1 | array_2G5 |
| p65 | 45 | 1 | 903787 | 1 | 1 | 1 | 1 | 1 | 220 | 1 | array_2A6 |
| p66 | 50.33 | 0 | 904163 | 1 | 1 | 0 | 1 | 1 | 210 | 1 | array_2C6 |
| p67 | 50.28 | 0 | 904371 | 1 | 1 | 0 | 1 | 1 | 288 | 1 | array_2D6 |
| p68 | 8.93 | 1 | 906084 | 0 | 0 | 0 | 2 | 1 | 206 | 0 | array_2E6 |
| p69 | 26.3 | 1 | 906246 | 1 | 1 | 0 | 1 | 0 | 82 | 0 | array_4B2 |
| p70 | 17.37 | 1 | 906357 | 1 | 1 | 1 | 3 | 0 | 92 | 0 | array_2G6 |
| p71 | 49.57 | 0 | 906435 | 1 | 1 | 1 | 1 | 1 | 296 | 1 | array_2B5 |
| p72 | 60 | 0 | 906439 | 0 | 0 | 0 | 3 | 1 | 294 | 1 | array_4C2 |
| p73 | 45 | 1 | 906472 | 1 | 1 | 0 | 3 | 1 | 292 | 1 | array_2F9 |
| p74 | 60 | 0 | 906952 | 0 | 0 | 0 | 2 | 1 | 291 | 1 | array_4D2 |
| p75 | 20.77 | 1 | 907126 | 0 | 1 | 0 | 1 | 1 | 166 | 0 | array_2B2 |
| p76 | 48.77 | 0 | 907306 | 1 | 0 | 0 | 2 | 1 | 247 | 1 | array_2C2 |
| p77 | 23 | 1 | 907407 | 1 | 1 | 0 | 2 | 0 | 112 | 1 | array_2D2 |
| p78 | 48.37 | 0 | 907862 | 1 | 1 | 0 | 1 | 1 | 183 | 0 | array_2E2 |
| p79 | 27.3 | 1 | 908201 | 1 | 1 | 1 | 1 | 1 | 154 | 0 | array_4E2 |
| p80 | 60 | 0 | 908705 | 0 | 0 | 0 | 1 | 0 | 31 | 1 | array_4G2 |
| p81 | 47.63 | 0 | 909400 | 0 | 1 | 0 | 2 | 1 | 217 | 1 | array_2A3 |
| p82 | 14.03 | 1 | 909461 | 1 | 0 | 1 | 3 | 1 | 208 | 1 | array_2B3 |
| p83 | 60 | 0 | 909596 | 0 | 1 | 0 | 1 | 1 | 220 | 1 | array_4A3 |
| p84 | 17.9 | 1 | 909687 | 1 | 0 | 0 | 1 | 0 | 89 | 0 | array_4B3 |
| p85 | 60 | 0 | 909761 | 1 | 0 | 1 | 1 | 0 | 0 | 1 | array_4C3 |
| p86 | 47.02 | 0 | 910090 | 1 | 1 | 0 | 1 | 1 | 278 | 0 | array_2C3 |
| p87 | 40 | 1 | 910399 | 0 | 1 | 0 | 3 | 1 | 224 | 0 | array_2D3 |
| p88 | 46.9 | 0 | 910757 | 1 | 1 | 0 | 1 | 1 | 282 | 1 | array_2E3 |
| p89 | 27.77 | 1 | 912061 | 1 | 0 | 0 | 2 | 1 | 281 | 1 | array_2G3 |
| p90 | 46.07 | 0 | 912333 | 1 | 1 | 0 | 2 | 1 | 290 | 0 | array_2A4 |
| p91 | 58.9 | 0 | 912723 | 0 | 0 | 0 | 1 | 1 | 177 | 1 | array_4D3 |
| p92 | 45.83 | 0 | 912773 | 1 | 1 | 1 | 1 | 1 | 152 | 1 | array_2B4 |
| p93 | 7.33 | 1 | 912938 | 1 | 1 | 0 | 3 | 1 | 161 | 0 | array_2C4 |
| p94 | 58.8 | 0 | 913139 | 0 | 0 | 0 | 1 | 1 | 188 | 1 | array_4E3 |
| p95 | 57.9 | 0 | 914552 | 1 | 1 | 0 | 1 | 0 | 88 | 1 | array_4G3 |
| p96 | 55.7 | 1 | 914808 | 0 | 0 | 0 | 2 | 1 | 230 | 0 | array_4B4 |
| p97 | 55.8 | 1 | 914869 | 1 | 1 | 0 | 2 | 1 | 297 | 0 | array_4C4 |
| p98 | 57.1 | 0 | 915847 | 0 | 0 | 0 | 1 | 1 | 189 | 1 | array_4F4 |
| p99 | 30.8 | 1 | 916200 | 0 | 1 | 0 | 1 | 0 | 0 | 0 | array_4G4 |
| p100 | 56.3 | 0 | 917072 | 1 | 1 | 0 | 1 | 1 | 300 | 1 | array_4A5 |
| p101 | 55.8 | 0 | 918173 | 1 | 1 | 0 | 1 | 1 | 184 | 1 | array_4C5 |
| p102 | 46.8 | 1 | 918215 | 1 | 1 | 0 | 1 | 0 | 91 | 0 | array_4D5 |
| p103 | 55.4 | 0 | 918782 | 0 | 0 | 0 | 3 | 1 | 278 | 1 | array_4E5 |
| p104 | 27.7 | 1 | 919402 | 1 | 0 | 0 | 1 | 0 | 82 | 0 | array_4F5 |
| p105 | 54.6 | 0 | 920136 | 1 | 1 | 0 | 1 | 0 | 93 | 1 | array_4G5 |
| p106 | 31.6 | 1 | 1000678 | 1 | 0 | 0 | 3 | 1 | 189 | 1 | array_4B6 |
| p107 | 53 | 0 | 1001519 | 0 | 1 | 0 | 1 | 1 | 231 | 1 | array_4C6 |
| p108 | 29 | 1 | 1001587 | 0 | 0 | 0 | 1 | 0 | 0 | 1 | array_4D6 |
| p109 | 24.8 | 1 | 1001962 | 1 | 0 | 0 | 2 | 0 | 91 | 0 | array_4E6 |
| p110 | 31.7 | 1 | 1002161 | 0 | 0 | 0 | 2 | 0 | 87 | 1 | array_4F6 |
| p111 | 52.6 | 0 | 1002277 | 0 | 0 | 0 | 1 | 1 | 161 | 1 | array_4G6 |
| p112 | 51.4 | 0 | 1003971 | 0 | 0 | 0 | 1 | 1 | 281 | 1 | array_4A7 |
| p113 | 50.4 | 0 | 1005962 | 0 | 0 | 0 | 1 | 1 | 188 | 0 | array_4E7 |
| p114 | 15.6 | 1 | 1006156 | 1 | 1 | 1 | 1 | 0 | 43 | 0 | array_4C7 |
| p115 | 36.3 | 1 | 1006518 | 1 | 1 | 0 | 1 | 0 | 0 | 0 | array_4D7 |
| p116 | 49.6 | 0 | 1007934 | 1 | 1 | 0 | 1 | 1 | 157 | 0 | array_4G7 |
| p117 | 27.9 | 1 | 1008700 | 1 | 1 | 1 | 1 | 0 | 69 | 1 | array_4C8 |
| p118 | 20.1 | 1 | 1009095 | 0 | 0 | 1 | 3 | 0 | 31 | 1 | array_4D8 |
| p119 | 9.8 | 1 | 1009740 | 1 | 0 | 0 | 1 | 1 | 279 | 1 | array_4E8 |
| p120 | 48.8 | 0 | 1009843 | 0 | 1 | 0 | 1 | 1 | 194 | 0 | array_4F8 |
| p121 | 48.5 | 0 | 1010270 | 0 | 0 | 0 | 3 | 1 | 193 | 1 | array_4G8 |
| p122 | 47.1 | 0 | 1013334 | 0 | 0 | 0 | 1 | 1 | 157 | 1 | array_4C10 |
| p123 | 46.9 | 0 | 1013632 | 1 | 0 | 0 | 1 | 1 | 216 | 1 | array_4D10 |
| p124 | 42.8 | 1 | 1013707 | 0 | 1 | 0 | 2 | 1 | 213 | 0 | array_4E10 |
| p125 | 18.8 | 1 | 1013841 | 0 | 1 | 0 | 2 | 0 | 113 | 0 | array_4F10 |
| p126 | 49.7 | 0 | 1014091 | 1 | 0 | 0 | 1 | 1 | 190 | 1 | array_6A1 |
| p127 | 8 | 1 | 1015368 | 1 | 1 | 1 | 2 | 1 | 162 | 0 | array_6C1 |
| p128 | 45.2 | 0 | 1016678 | 0 | 1 | 0 | 1 | 1 | 160 | 0 | array_6F1 |
| p129 | 45.1 | 0 | 1017023 | 0 | 1 | 0 | 1 | 0 | 109 | 1 | array_6G1 |
| p130 | 45.1 | 0 | 1017090 | 0 | 1 | 0 | 1 | 0 | 55 | 1 | array_6A2 |
| p131 | 45 | 0 | 1017317 | 0 | 0 | 0 | 1 | 1 | 155 | 1 | array_6B2 |
| p132 | 30.3 | 1 | 1018487 | 1 | 0 | 1 | 1 | 1 | 229 | 1 | array_6C2 |
| p133 | 44.3 | 0 | 1018499 | 1 | 1 | 1 | 1 | 1 | 189 | 1 | array_6D2 |
| p134 | 44.2 | 0 | 1018837 | 0 | 1 | 0 | 2 | 1 | 206 | 0 | array_6E2 |
| p135 | 39 | 1 | 1019278 | 1 | 0 | 0 | 1 | 0 | 103 | 1 | array_6F2 |
| p136 | 43.5 | 0 | 1020254 | 0 | 0 | 0 | 1 | 1 | 154 | 1 | array_6G2 |
| p137 | 28 | 1 | 1021366 | 1 | 1 | 0 | 1 | 0 | 106 | 0 | array_6B3 |
| p138 | 42.6 | 0 | 1022340 | 0 | 1 | 0 | 1 | 1 | 291 | 0 | array_6C3 |
| p139 | 42.3 | 0 | 1022711 | 1 | 0 | 1 | 1 | 1 | 156 | 1 | array_6D3 |
| p140 | 40.8 | 1 | 1023168 | 0 | 0 | 0 | 1 | 1 | 211 | 0 | array_6A4 |
