# Supplementary figures and images for: Loss of IL-34 Expression Indicates Poor Prognosis in Patients With Lung Adenocarcinoma
Source: Front Oncol. 2021 Jul 16;11:639724. doi: 10.3389/fonc.2021.639724 (PMC8322957; doi:10.3389/fonc.2021.639724)

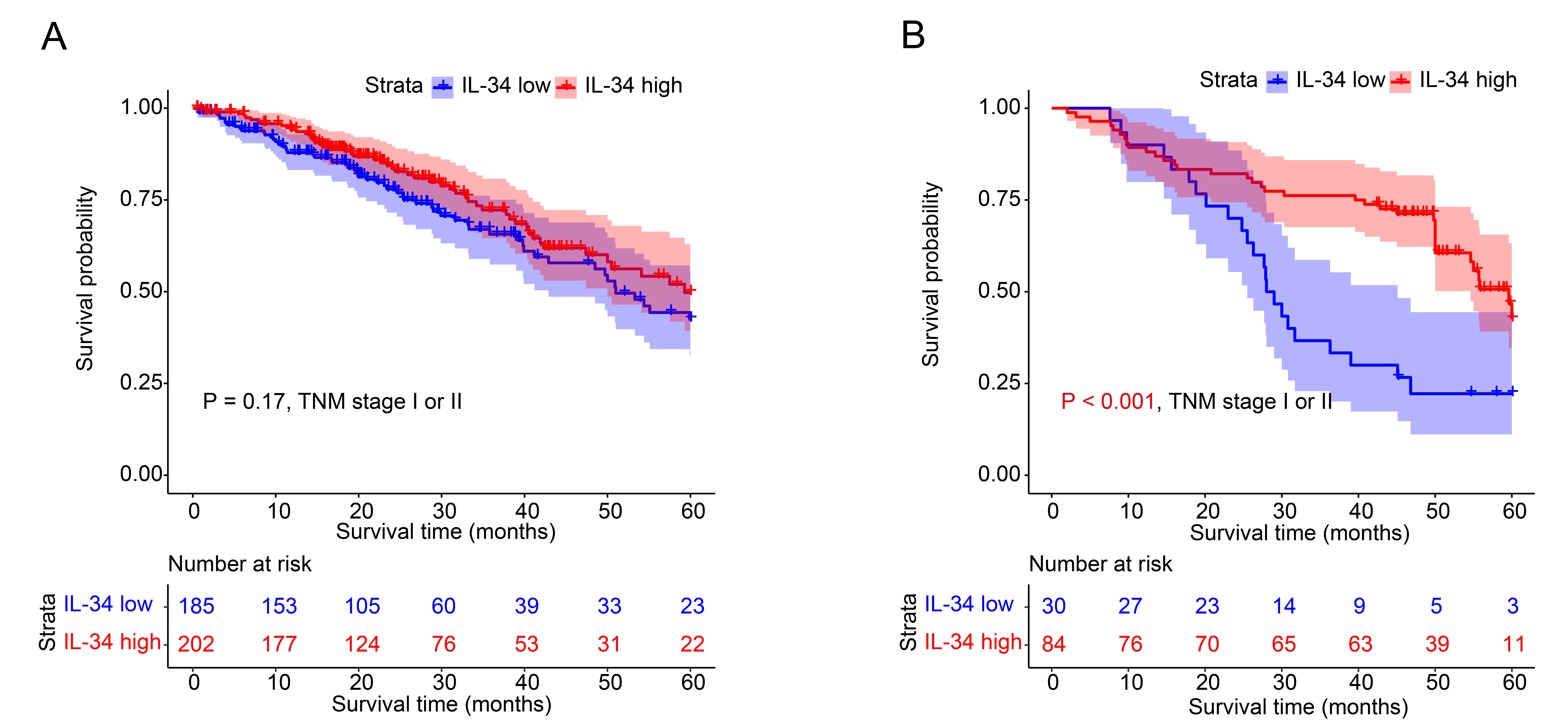

Supplement: Supplementary file 2 [file Image_1.jpeg]

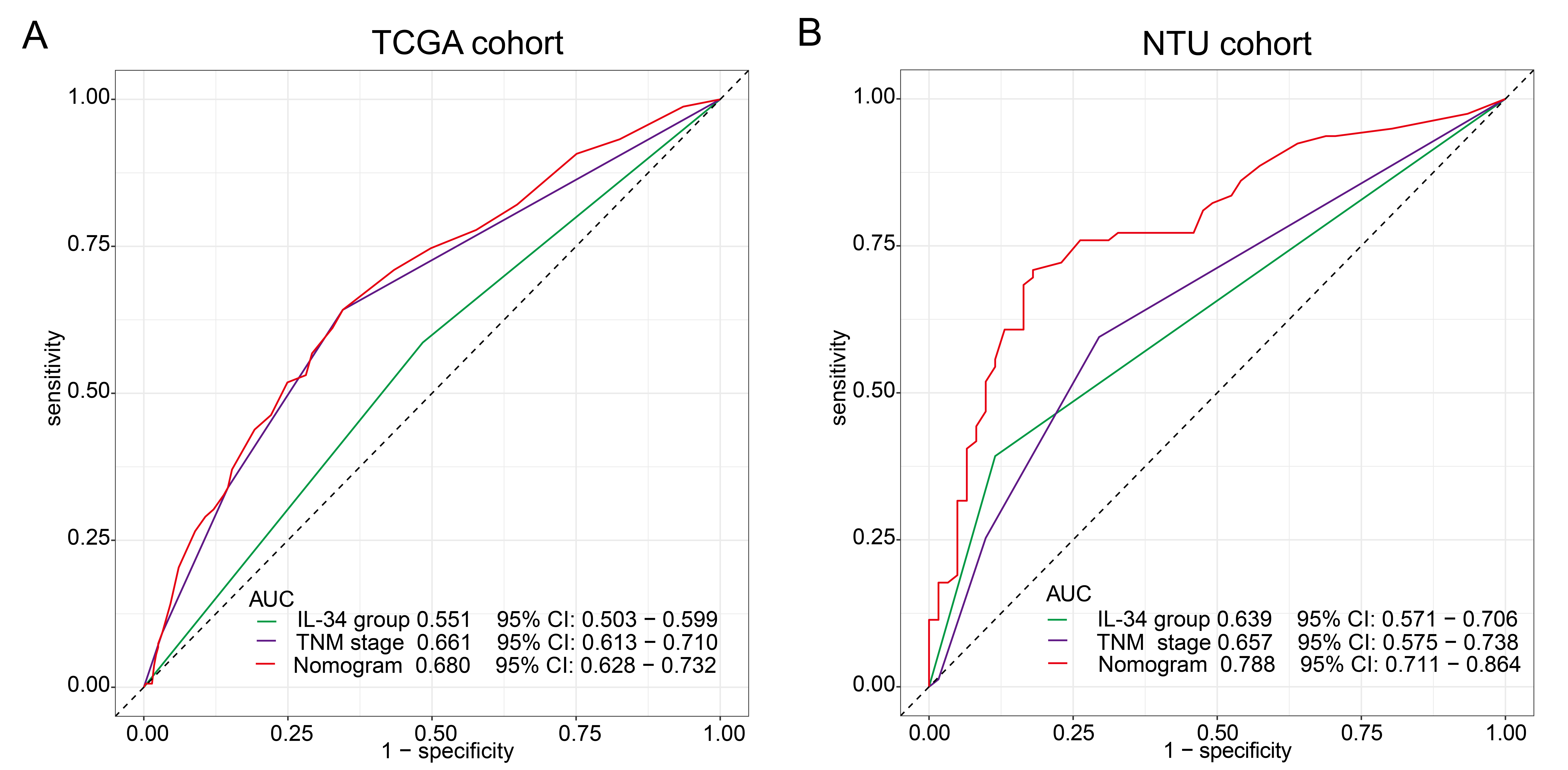

Supplement: Supplementary file 4 [file Image_3.jpeg]
